# Current Tolerance-Associated Peripheral Blood Gene Expression Profiles After Liver Transplantation Are Influenced by Immunosuppressive Drugs and Prior Cytomegalovirus Infection

**DOI:** 10.3389/fimmu.2021.738837

**Published:** 2022-01-11

**Authors:** Aafke A. Duizendstra, Michelle V. van der Grift, Patrick P. Boor, Lisanne Noordam, Robert J. de Knegt, Maikel P. Peppelenbosch, Michiel G. H. Betjes, Nicolle H. R. Litjens, Jaap Kwekkeboom

**Affiliations:** ^1^ Department of Gastroenterology and Hepatology, Erasmus MC University Medical Center, Rotterdam, Netherlands; ^2^ Erasmus MC Transplant Institute, Division of Nephrology and Transplantation, Department of Internal Medicine, Erasmus MC University Medical Center, Rotterdam, Netherlands

**Keywords:** liver transplantation, operational tolerance, peripheral blood gene expression, cytomegalovirus infection, immunosuppressive drugs

## Abstract

Spontaneous operational tolerance to the allograft develops in a proportion of liver transplant (LTx) recipients weaned off immunosuppressive drugs (IS). Several previous studies have investigated whether peripheral blood gene expression profiles could identify operational tolerance in LTx recipients. However, the reported gene expression profiles differed greatly amongst studies, which could be caused by inadequate matching of clinical parameters of study groups. Therefore, the purpose of this study was to validate differentially expressed immune system related genes described in previous studies that identified tolerant LTx recipients after IS weaning. Blood was collected of tolerant LTx recipients (TOL), a control group of LTx recipients with regular IS regimen (CTRL), a group of LTx recipients with minimal IS regimen (MIN) and healthy controls (HC), and groups were matched on age, sex, primary disease, time after LTx, and cytomegalovirus serostatus after LTx. Quantitative Polymerase Chain Reaction was used to determine expression of twenty selected genes and transcript variants in PBMCs. Several genes were differentially expressed between TOL and CTRL groups, but none of the selected genes were differentially expressed between HC and TOL. Principal component analysis revealed an IS drug dosage effect on the expression profile of these genes. These data suggest that use of IS profoundly affects gene expression in peripheral blood, and that these genes are not associated with operational tolerance. In addition, expression levels of SLAMF7 and NKG7 were affected by prior cytomegalovirus infection in LTx recipients. In conclusion, we found confounding effects of IS regimen and prior cytomegalovirus infection, on peripheral blood expression of several selected genes that were described as tolerance-associated genes by previous studies.

## Introduction

For end-stage liver disease a liver transplantation (LTx) is the sole treatment option. Since long-term use of immunosuppressive drugs (IS) could lead to several serious side effects and adversely impacts quality of life after transplantation, most transplantation centers attempt to gradually reduce or even completely wean IS over time (1–4). Several clinical trials have shown that some LTx recipients can develop operational tolerance towards their graft, a long-term state where (acute) rejection episodes are absent after IS are fully weaned (5–7).

In the last fifteen years considerable efforts have been made to identify noninvasive biomarkers of operational tolerance in LTx. Several studies have investigated whether tolerant LTx recipients could be discriminated from a control group or a non-tolerant group of LTx recipients with regular IS regimen by examining gene expression in circulating peripheral blood mononuclear cells (PBMCs) (8–13). Herein it was suggested that certain gene profiles related to the general immune system, natural killer (NK) cells, γδT-cells and regulatory T-cells (Tregs) could identify tolerant LTx recipients. Strikingly however, these gene profiles differed greatly amongst these studies. Several reasons may account for these differences. Firstly, in all studies, except the study of Bohne et al. (8), gene expression profiles of tolerant LTx recipients without IS regimen were compared to control or non-tolerant LTx recipients with IS regimen (9–13). Therefore, gene expression profiles in the control or non-tolerant LTx recipients may have been affected by IS. Furthermore, thorough matching of parameters known to influence immune cell composition and gene expression, such as age, sex, IS usage, (viral) primary disease and prior cytomegalovirus (CMV) infection, between study groups was not performed. CMV infection constitutionally inflates memory(like) peripheral T-cell and NK cell compartments and circulating γδT-cells (14, 15). In addition, in kidney and liver Tx recipients with regular IS regimen a durable change in the circulating immune cell composition was observed after CMV infection (16–21). Moreover, afore mentioned studies have used microarray and/or polymerase chain reaction (PCR) to study gene expression, but it is unclear which splice variants of the studied genes have been detected.

Therefore, the purpose of this study was to validate previously reported transcriptional profiles of immune system related genes in peripheral blood of tolerant LTx recipients. Validation was performed by comparing peripheral blood gene expression profiles of tolerant LTx recipients without IS, a control group of LTx recipients with regular IS regimen, a group of LTx recipients with minimal IS regimen to reveal possible effects of IS, and healthy controls. These groups were matched for important parameters known to influence immune cell composition and their gene expression in peripheral blood.

## Patients And Methods

### Study Design and Participants

In this study blood samples were collected from three different groups of adult LTx recipients late after LTx and an adult healthy control group. A group of operational tolerant LTx recipients (TOL; n=13) that were followed at the outpatient clinic at the Erasmus University Medical Center between 2014 and 2020 was included. TOL were completely weaned off IS for medical reasons or non-compliance between 2008 and 2019 and did not experience acute rejection. Acute rejection was defined as at least a two-fold increase in serum bilirubin, aspartate aminotransferase or alanine transaminase, alkaline phosphatase or γ-glutamyltransferase, that normalized upon adequate IS regimen. Protocol biopsies after complete IS weaning were not taken because of possible complications related to the procedure. In five tolerant LTx recipients a liver biopsy was performed because of possible rejection as indicated by increasing liver enzymes, at on average 3.1 ± 2.2 years after complete weaning. Rejection was excluded according to BANFF criteria. A control group of stable LTx recipients (CTRL; n=24) with regular dual or mono IS regimen and a group of stable LTx recipients (MIN; n=8) with minimal mono IS regimen were also included. These groups were matched to the TOL group for important parameters known to influence circulating immune cells (**Table 1**). IS mono therapy trough levels for CTRL were Tacrolimus 3.2-7.8 μg/L, Mycophenolate Mofetil >2.9 mg/L, and for MIN were Tacrolimus 1.2-2.5 μg/L, Cyclosporin A 58 μg/L, Mycophenolate Mofetil 0.9 mg/L. Both CTRL and MIN did not experience rejection episodes for at least 5 years before and 4 years after blood collection. Immunoglobulin G (IgG) antibodies to CMV in serum were measured with an enzyme immune assay (Biomerieux, VIDAS, Lyon, France). An outcome of ≥6 AU/mL was considered positive. A matched healthy control group (HC; n=7) was also included in the study. Clinical and laboratory information was retrieved from electronic patient records. Informed consent was received from all participants. This study was conducted in accordance with the Declaration of Helsinki and approved by the medical ethics committee of Erasmus MC (MEC 2014-232; MEC 2012-022).

**Table 1:** Characteristics of the study groups.

	CTRL	MIN	TOL	HC	*P-value*
**Demographics**	n=24	n=8	n=13	n=7	
Male (%)	62.5	50.0	76.9	71.4	0.43
Age in years^a^	55.0 (30.5-58.5)	57.5 (37.8-64.8)	56.0 (43.0-68.5)	45.0 (25.0-54.0)	0.56
Years post-LTx^a^	14.5 (12.0-20.5)	15.5 (12.3-18.8)	15.0 (13.0-17.5)	NA	0.83
Years complete weaning - end follow-up^a^	NA	NA	4.0 (2.0-6.0)	NA	NA
**Primary disease (%)**				NA	0.61
Cholestatic disease	25.0	12.5	30.8		
Virus-related^b^	33.3	75.0	30.8		
Hepatocellular carcinoma	20.8	0.0	23.1		
Cryptogenic cirrhosis	12.5	0.0	15.4		
Toxicity-induced	4.2	0.0	0.0		
Metabolic-related	4.2	0.0	0.0		
Rupture	0.0	12.5	0.0		
**IS (last) used (%)**				NA	0.45
Tac	66.7	75.0	53.8		
CsA	4.2	12.5	7.7		
MMF	8.3	12.5	0.0		
Aza	0.0	0.0	7.7		
Tac and MMF	8.3	0.0	15.4		
Pred and Tac	8.3	0.0	0.0		
Pred and MMF	4.2	0.0	0.0		
Aza and CsA	0.0	0.0	7.7		
Unknown	0.0	0.0	7.7		
**CMV seropositive (%)**				ND	
Recipient pre-LTx	50.0	50.0	46.2		0.92
Recipient post-LTx	75.0	87.5	61.5		0.78
Donor	45.8	50.0	46.2		0.85

Percentages or ^a^median years with 25th and 75th IQR are presented. Statistical analyses were performed with Chi-Square or Kruskal-Wallis rank test. ^b^Viral-related liver diseases include Hepatitis A, B or C virus, and Epstein Barr virus. Aza, azathioprine; CMV, cytomegalovirus; CsA, cyclosporine A; CTRL, control group; HC, healthy controls; IS, immunosuppressive drugs; LTx, liver transplantation; MIN, minimal IS group; MMF, mycophenolate mofetil; NA, not applicable; ND, not determined; Pred, prednisolone; Tac, tacrolimus; TOL, tolerant group.

### Primers

Twenty-two immune system-related candidate genes (KLRB1, CD160, KLRC4, KLRF1, NKG7, IL2RB, IRF5, EGR2, CXCL8, ZBTB21, CX3CR1, OSBPL5, SLAMF7, ERBB2, UBD, FOXP3, SMAD2, SMAD3, TET1, TET2, HELIOS, NRP1) that have shown differential expression in TOL LTx recipients versus a control or non-tolerant group of LTx recipients in previous studies were selected (8–13). Forward and reverse primers were designed with NCBI PrimerBLAST according to MIQE guidelines (22). To prevent co-amplification of genomic DNA, intron-flanking primers or exon-exon junction primers were designed with target amplicon sequences of 80 to 150 bp with a maximum GC content of 65%. Forward and reverse primers were not modified and were purified with a desalt step (Merck, Darmstadt, Germany). Primer pairs were tested in duplicate for their optimal annealing temperature and amplification efficiency using healthy control PBMC derived cDNA. Four temperatures were tested to determine the optimal annealing temperature (56, 58, 60, 62°C) for each primer pair. Amplification efficiency was tested with a serial dilution series and efficiencies within the range of 90–110% were considered acceptable. Gel electrophoresis was performed to detect presence of unintended target amplicon sequences using a 2% agarose gel (Merck) with 10% TBE buffer (Thermo Scientific, Waltham, USA) and 1:100.000 DNA Stain G (SERVA, Heidelberg, Germany). Size of the PCR product was determined with a six times dilution using Blue/Orange Loading Dye (Promega, Madison, USA) and a 25bp DNA Step ladder (Promega), and compared with intended target amplicon sequence size to confirm specificity of the primer pair. Primer pairs for ERBB2 and UBD did not pass all tests, even after multiple attempts of re-designing primer pairs. Primer pairs for the remaining twenty selected genes and three housekeeping genes passed all tests and are presented in **Supplementary Table 1**. Most primer pairs targeted all splicing and transcript variants of the selected gene. Some selected genes required design of multiple primer pairs to target multiple splice variants (NKG7, ZBTB21, CX3CR1 and SLAMF7). For CX3CR1 and KLRF1 not all splice variants could be detected with selected primer pairs.

### RNA Isolation and Generation of cDNA

Peripheral blood mononuclear cells (PBMCs) were isolated using Ficoll density gradient centrifugation (GE Healthcare, Little Chalfont, England), and were stored in RA1 lysis buffer (MACHEREY-NAGEL, Dueren, Germany) and β-Mercaptoethanol (Merck) at a concentration of 3x10^6^ cells at -80°C until further use. RNA was isolated with NucleoSpin RNA Mini (MACHEREY-NAGEL) according to standard protocol that includes a DNase step. Purity and quantity of isolated RNA was measured with NanoDrop (Thermo Scientific). A 260nm/280nm ratio of ~2.0 was considered pure RNA. cDNA was generated in 50μl (36ng/μl) using 5x PrimeScript™ RT Master Mix Perfect Real Time (Takara, Shiga, Japan) and SimpliAmp Thermal Cycler (Thermo Scientific). Concentration of cDNA was set to 2.5 ng/μl and stored at 4°C until short-term further use.

### Quantitative Polymerase Chain Reaction

Gene expression was determined in triplicate with 12.5ng cDNA/reaction using SYBR Select Master Mix for CFX (Thermo Scientific) measured by StepOnePlus Real-Time PCR System (Applied Biosystems Thermo Scientific) and analyzed with StepOne software version 2.3 (Applied Biosystems Thermo Scientific). Thermocycling parameters include a UDG activation step at 50°C for 2 min and AmpliTaq DNA Polymerase and UP Activation step at 95°C for 2 min. Thereafter, 40 PCR cycles with a denaturing step at 95°C for 15 sec, the determined optimal annealing temperature for 15 sec and extension at 72°C for 1 min continued. Thermocycling ended with a meltcurve stage with 95°C for 15 sec, 60°C for 1 min, after which an increase of 0.3°C/2sec to 95°C occurred. Gene expression was considered positive when <35 cycles were needed to detect a signal. After each measurement the meltcurve was examined to confirm exclusive amplification of the intended target gene. Expression of twenty selected genes was normalized with mean expression of three housekeeping genes (GAPDH, GUSB, and HPRT1; **Supplementary Table 1**) using the comparative Ct method.

### Statistical Analyses

Statistical analyses were performed with IBM SPSS software version 25 (SPSS Inc., Chicago, USA) or GraphPad Prism 8 version 8.4.3 (GraphPad Software Inc., San Diego, USA). The normality of the distribution of the data was determined by the Shapiro-Wilk normality test. Statistical analyses were performed with one-way ANOVA or Kruskal-Wallis, with a Bonferroni or Dunn’s posttest. Differences in discrete nominal data between groups were analyzed by the Pearson Chi-Square test. Figures and heatmap were created with GraphPad Prism 8 version 8.4.3. Principal component analysis using direct oblimin factor rotation was performed using IBM SPSS software version 25.

## Results

### Patient Characteristics

In this study operationally tolerant (TOL) LTx recipients were compared to a group of control (CTRL) LTx recipients with regular IS regimen, a group of LTx recipients with minimal IS monotherapy regimen (MIN) and a group of healthy controls (HC) (**Table 1**). These groups were all carefully matched for important parameters known to influence expression of immune system related genes. Therefore, the study groups did not differ in age, sex, time after transplantation, primary liver disease, and CMV serostatus of the donor and recipient before and after (at the time of blood collection) transplantation. Although the groups did not significantly differ in primary liver disease, the MIN LTx recipients harbor the highest prevalence of virus-related liver disease before LTx.

### Use of Immunosuppressive Drugs Affects Gene Expression of PBMCs

**Figure 1:**
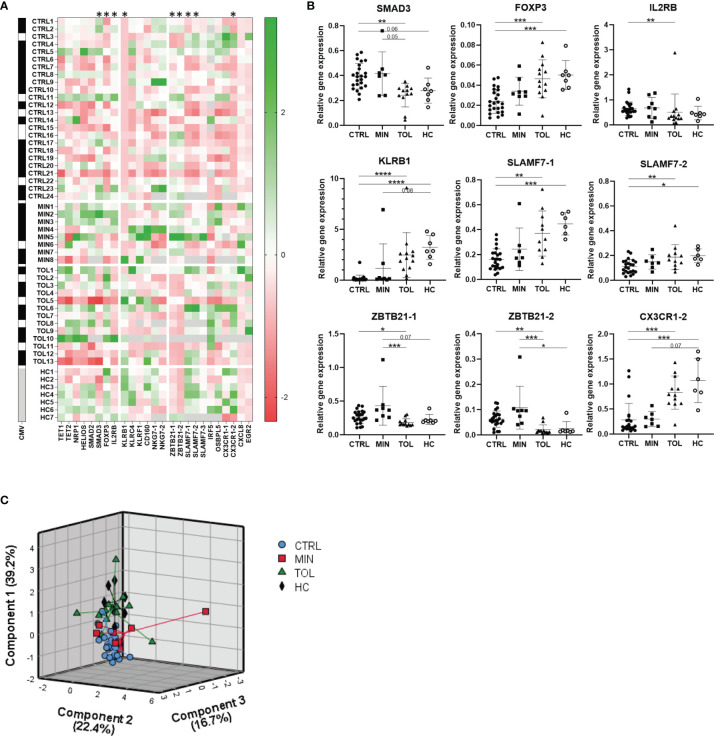
Use of immunosuppressive drugs affects gene expression. **(A)** A heatmap is presented with Z-scores derived from relative expression of each gene compared to its mean expression in all subjects. Green squares indicate an upregulation and red squares indicate a downregulation compared to the mean of the indicated gene. On the left black squares indicate CMV seropositivity, whereas white squares indicate CMV seronegativity for each study subject. Grey squares indicate that gene expression or CMV seropositivity was not determined. The annotation with -1 or -2 indicate that different splice variants of that gene are included. * Relative gene expression of indicated gene was significantly different between TOL and CTRL LTx recipients. **(B)** The nine significant differentially relatively expressed genes between TOL and CTRL are presented. The annotations with -1 or -2 indicate that different splice variants of that gene are included. *P < 0.05, **P < 0.01, ***P < 0.001, ****P < 0.0001 (derived with Bonferroni or Dunn’s posttest) **(C)** Principal component analysis of all study groups with the nine significant differentially expressed genes between TOL and CTRL LTx recipients is depicted. Rotated component matrix analysis was performed using direct oblimin factor rotation. On the axes the contributed percentage of the variance between groups by that component is indicated. CMV, cytomegalovirus; CTRL, control LTx recipients; HC, healthy control; LTx, liver transplantation; MIN, minimal IS regimen LTx recipients; TOL, tolerant LTx recipients.

Relative gene expression of PBMCs was assessed for twenty genes and their transcript variants (annotation -1, -2 or -3) that were selected from previous studies in which their expression level was reported to be associated with operational tolerance after LTx (**Supplementary Table 1** and **Figure 1A**). Expression of TET1, TET2, NRP1, HELIOS, NKG7-1, NKG7-2, IRF5, EGR2, OSBPL5 and CX3CR1-1 genes did not differ among groups (**Figure 1A**). Expression of KLRC4 and SLAMF7-3 was significantly higher in HC compared to CTRL, but did not differ between other groups (**Figure 1A** and **Supplementary Figure 1**). Gene expression of KLRF1, SMAD2, CXCL8 and CD160 was significantly higher in MIN compared to TOL, CTRL and/or HC but did not differ between TOL and CTRL groups (**Figure 1A** and **Supplementary Figure 1**). This data might imply that expression of KLRF1, SMAD2, CXCL8, and CD160 could have been influenced by viral infections, since the majority of MIN LTx recipients were transplanted for virus-related primary liver disease. Gene expression of SMAD3, FOXP3, IL2RB, KLRB1, SLAMF7-1, SLAMF7-2, ZBTB21-1, ZBTB21-2 and CX3CR-2 was significantly different in TOL compared to CTRL (**Figures 1A, B**). Of these genes, expression of FOXP3, KLRB1, SLAMF7-1, SLAMF7-2 and CX3CR-2 also significantly differed between HC and CTRL. However, none of the twenty selected genes significantly differed between TOL and HC. This indicates that the differential expression of the genes between TOL and CTRL does not represent a tolerance-associated gene profile, but rather reflects a difference in IS usage. Principal component analysis of the nine significantly different expressed genes between TOL and CTRL revealed three components that separated CTRL from clustered HC and TOL, with MIN clustered in-between the groups (**Figure 1C**). This suggests that gene expression of SMAD3, FOXP3, IL2RB, KLRB1, SLAMF7-1, SLAMF7-2, ZBTB21-1, ZBTB21-2 and CX3CR-2 in CTRL and MIN LTx recipients is affected by the height of the IS through levels. This is most clearly observed in the stepwise increase of FOXP3, KLRB1, and SLAMF7-1 expression from CTRL to MIN to TOL and HC (**Figure 1B**).

### Prior CMV Infection Affects Gene Expression of PBMCs

**Figure 2:**
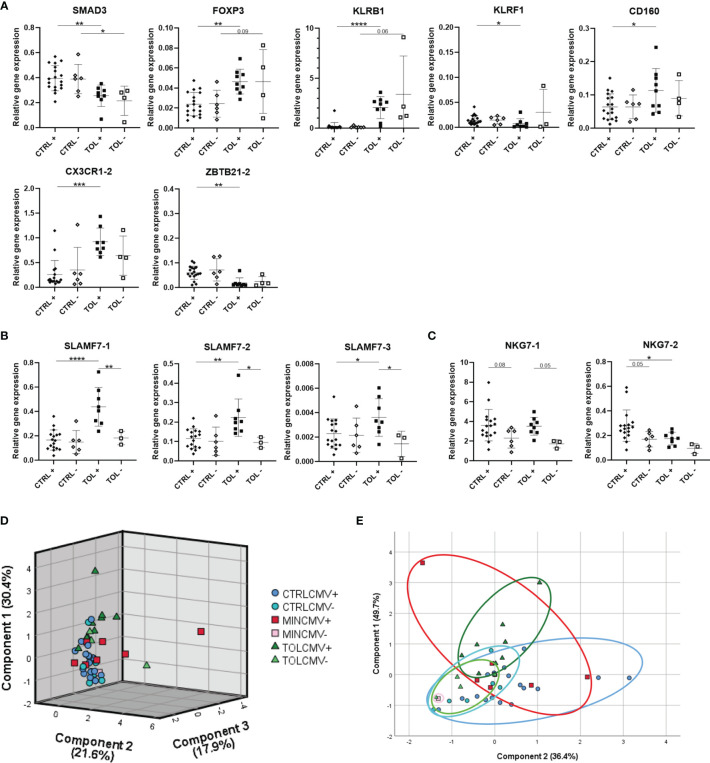
Prior cytomegalovirus infection affects gene expression of PBMCs. **(A)** Seven significant differentially expressed genes between CMV seropositive TOL and CTRL LTx recipients are presented. **(B)** Differentially expressed SLAMF7 splice variants in CMV seropositive TOL are depicted. **(C)** Tendencies to differential expression of NKG7 splice variants in CMV seropositive TOL and CTRL are depicted. **(D)** Principal component analysis of expression of the eleven gene variants which were significantly differentially expressed between CMV seropositive TOL and CTRL LTx recipients is depicted. **(E)** Principal component analysis of splice variants of SLAMF7 and NKG7 presented in **(B, C)** is depicted. **(D, E)** Rotated component matrix analysis was performed using direct oblimin factor rotation. On the axes the contributed percentage of the variance between groups by that component is indicated. The annotation with -1 or -2 indicates that different splice variants of that gene are included. + indicates CMV seropositivity, - indicates CMV seronegativity. *P < 0.05, **P < 0.01, ***P < 0.001, ****P < 0.0001 (derived with Bonferroni or Dunn’s posttest). CMV, cytomegalovirus; CTRL, control LTx recipients; HC, healthy control; LTx, liver transplantation; MIN, minimal IS regimen LTx recipients; TOL, tolerant LTx recipients.

Our study groups were all carefully matched for important parameters known to influence expression of immune system related genes, such as prior CMV infection. To study the influence of prior CMV infection on gene expression, TOL and CTRL LTx recipients were divided according to their CMV serostatus at the time of blood collection late after LTx (**Figure 2**). Expression of SMAD3, FOXP3, KLRB1, KLRF1, CD160, CX3CR1-2 and ZBTB21-2 in CMV seropositive TOL differed (significantly) from CMV seropositive CTRL LTx recipients (**Figure 2A**). Expression of SMAD3 did significantly differ between TOL and CTRL CMV seronegative LTx recipients. Expression of other genes did not significantly differ between TOL and CTRL CMV seronegative LTx recipients, which is probably due to the low number of CMV seronegative individuals. These results could indicate that these genes are influenced by the use of IS, but not prior CMV infection. In contrast, gene expression of SLAMF7-1, SLAMF7-2 and SLAMF7-3 splice variants were significantly higher in CMV seropositive TOL compared to CMV seropositive CTRL and CMV seronegative TOL (**Figure 2B**). Expression of NKG7-1 tended to be higher in CMV seropositive TOL and CTRL versus their CMV seronegative counterpart (**Figure 2C**). Expression of NKG7-2 splice variant tended to be higher in CMV seropositive CTRL compared to CMV seronegative CTRL and was significantly higher in CMV seropositive CTRL compared to CMV seropositive TOL. These data show that prior CMV infection is associated with a higher relative gene expression of SLAMF7 and NKG7 in PBMCs of LTx recipients, but the increase in SLAMF7 expression in CMV seropositive CTRL was hampered by the use of IS. Principal component analysis of the eleven significantly different expressed genes and transcript variants between CMV seropositive TOL and CTRL revealed three components that separated CTRL from TOL, with most MIN in-between (**Figure 2D**). Strikingly, the CMV seronegative individuals positioned generally below the seropositive individuals for all three groups. Principal component analysis of the five SLAMF7 and NKG7 splice variants, which expression was influenced by prior CMV infection, revealed two components (**Figure 2E**). In this analysis the CMV seronegative LTx recipients clustered completely together, whereas CMV seropositive TOL and CTRL clustered partly separately with overlap of CMV seropositive MIN, indicative of an IS and a prior CMV infection effect on gene expression in PBMCs of these groups.

### Full Disclosure of Analyzed Splice Variants of Genes Is Necessary

**Figure 3:**
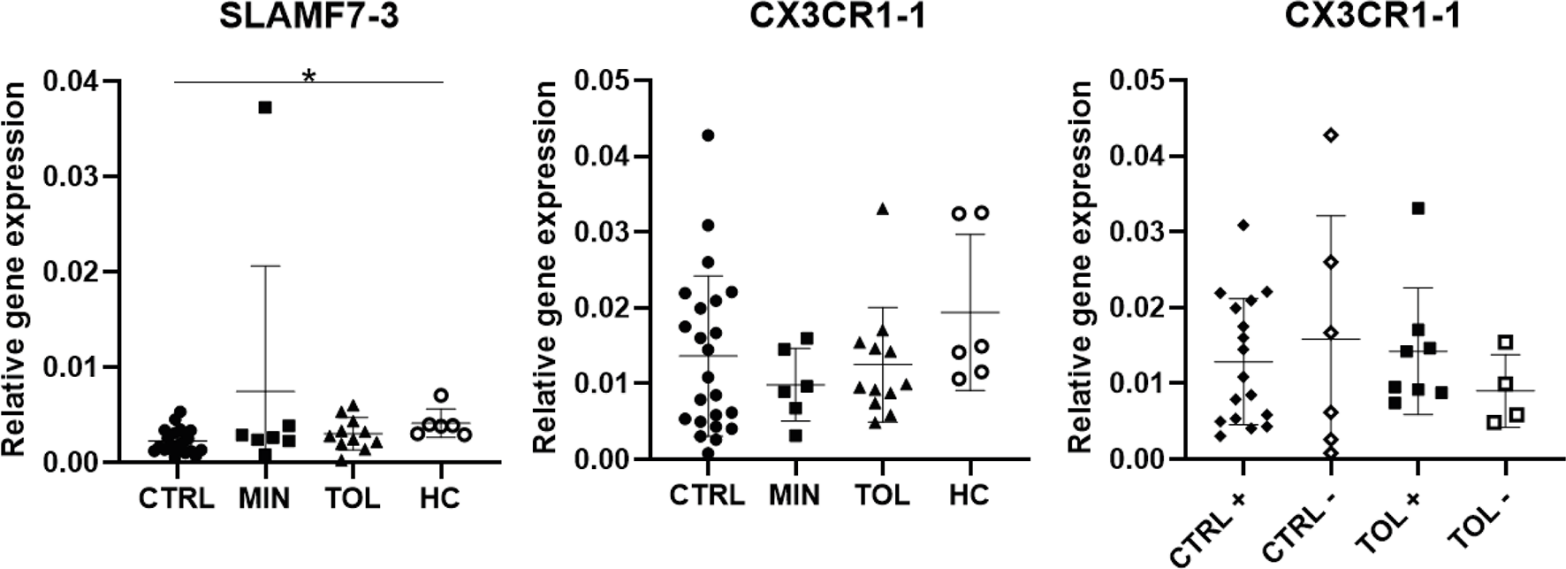
Differential splice variant expression of selected genes. Splice variants SLAMF-3 and CX3CR1-1 differ in their gene expression from splice variants SLAMF-1, SLAMF7-2 and CX3CR1-2. The annotation with -1 or -2 indicate that different splice variants of that gene are included. + indicates CMV seropositivity, - indicates CMV seronegativity. *P < 0.05 CMV, cytomegalovirus; CTRL, control LTx recipients; HC, healthy control; LTx, liver transplantation; MIN, minimal IS regimen LTx recipients; TOL, tolerant LTx recipients.

In our study we carefully presented the splice variants of the selected and analyzed genes (**Supplementary Table 1**). It appeared that expression of CX3CR1-1 and CX3CR1-2, SLAMF7-1 – 3, and NKG7-1 and NKG7-2 (**Figures 1B**, **2A, C** and **3**) splice variants were not similar to each other. Expression of SLAMF7-1 and SLAMF7-2, but not SLAMF7-3 (**Figure 3**), splice variants were significantly higher in TOL versus CTRL. Expression of CX3CR1-2 was significantly higher in TOL compared to CTRL, whereas CX3CR1-1 expression was not. Similarly, expression of CX3CR1-2 was significantly higher in CMV seropositive TOL compared to CMV seropositive CTRL, but not for CX3CR1-1 expression. These results indicate that it is important to always check and provide the analyzed data with splice variants for maximum transparency.

## Discussion

Here we studied peripheral blood expression of twenty different immune system related genes described in previous studies suitable for identification of tolerant LTx recipients. These genes include KLRB1, KLRC4, KLRF1, CD160, NKG7, FOXP3, IL2RB, SMAD2, SMAD3, TET1, TET2, HELIOS and NRP1, IRF5, EGR2, CXCL8, ZBTB21, CX3CR1, OSBPL5 and SLAMF7 (8–13). Our study indicates that previously reported differential expressions of these genes between tolerant and non-tolerant LTx recipients may have been profoundly influenced by differences in IS regimen, prior CMV infection, and potentially other differences between the study groups.

In our study expression of NKG7, IRF5, EGR2, OSBPL5, CX3CR1-1, TET1, TET2, NRP1 and HELIOS did not differ among groups. We performed our study using carefully matched study groups for important clinical demographics and characteristics known to influence expression of immune system related genes. The afore-mentioned studies lack thorough matching of such parameters. In the studies of Martínez-Llordella et al., Martínez-Llordella et al., Pons et al., Lozano et al., Bohne et al., and Revilla-Nuin et al. (8–13) age, sex, primary liver disease, time after LTx, and prior CMV infection, were either not described or greatly differed between study groups. These parameters could all have had a confounding effect on the reported differential gene expression between the study groups, and could explain the discrepancies observed with our study. This is also supported by the notion that in all of the above mentioned studies different gene expression profiles, with little common genes, were found that supposedly identified tolerant LTx recipients. That matching of clinical parameters is important is also illustrated by our data, where significantly higher expression levels of KLRF1, SMAD2, CXCL8, CD160 and ZBTB21 in the MIN group were observed, the LTx recipients that harbored the highest prevalence of viral liver disease before LTx. That viral liver diseases are capable of influencing peripheral blood gene expression is illustrated by Martínez-Llordella et al., 2007 and Martínez-Llordella et al., 2008, where Hepatitis C infection affected expression of many analyzed genes in tolerant, control and non-tolerant LTx recipients (10, 11). Another possible explanation for the discrepancies observed is that, in contrast to our study, three of the above mentioned studies (8, 10, 11) did not apply a correction factor for multiple statistical testing for data analysis and possibly have found statistical differences by chance. For a few genes, the discrepancies observed between different studies could also be due to assessment of different splice variants of the genes assessed. Unfortunately, it has not been reported which splice variants were analyzed in previously mentioned studies. That there are differences between expression of splice variants of several genes was illustrated by our own data, as well as those by others (23). Therefore, it is important to always provide data concerning the analyzed splice variants for full disclosure and maximum transparency when publishing research.

In our study gene expression of SMAD3, FOXP3, IL2RB, KLRB1, SLAMF7-1, SLAMF7-2, ZBTB21-1, ZBTB21-2 and CX3CR1-2 did significantly differ in TOL compared to CTRL LTx recipients. However, none of these genes significantly differed in expression between TOL and HC, suggesting an influence of IS. Principal component analysis revealed that the transcriptional profiles of these genes of MIN LTx recipients clustered in between CTRL and both the TOL and HC groups. This suggests that even the height of the IS trough levels affects expression of these genes, which was clearly observed for FOXP3, KLRB1 and SLAMF7-1. This could explain the differential expression between tolerant LTx recipients off IS and other LTx recipients on IS found in other studies. That IS influences expression levels of the studied genes is also supported, but definitely not clearly stated, by the study of Bohne et al. (8), where microarray analysis of tolerant and non-tolerant LTx recipients before prospective IS weaning resulted in a different tolerance associated gene profile in PBMCs compared to their own previous data on tolerant LTx recipients after IS weaning (9–11). KLRB1, SLAMF7, and CX3CR1 genes, of which the expression levels we found to be suppressed by IS, were reported among tolerance-associated genes in peripheral blood in previous studies, but not in the prospective weaning study (8). Moreover, one report admitted that peripheral blood gene expression patterns of TOL recipients without IS regimen appeared to be closer to those of healthy individuals than to those of non-TOL recipients with IS regimen (10). In addition, other studies have also indicated that use of tacrolimus affects gene expression in PBMCs of kidney and liver transplant recipients (24, 25). Therefore, we suggest that future studies on tolerance-associated genes in peripheral blood should be performed before IS weaning in LTx recipients.

Several studies have suggested that circulating NK cells and γδT-cells are implicated in operational tolerance (10, 11, 26). One study by Martínez-Llordella et al. (10) even suggested that three different gene profiles, including the mainly NK cell and γδT-cell related KLRF1, KLRB1, IL2RB, SLAMF7, NKG7 and CX3CR1 genes, in different combinations, could discriminate tolerant from non-tolerant LTx recipients after IS weaning (10). In our study we found that expression of KLRB1, IL2RB, SLAMF7 and CX3CR1 is probably affected by use of IS. Moreover, we found that prior CMV infection was associated with a higher relative gene expression of SLAMF7 and NKG7. It is known that CMV infection in healthy subjects changes the composition of circulating immune cells with expansion of pathogen-specific CD8+ T-cells, γδT-cells and NK cell subsets (27). CMV infection after kidney and liver transplantation induces similar long-lasting changes in these immune subsets (16–19, 21, 27, 28). As mentioned before, we carefully matched for demographical and clinical parameters, such as prior CMV infection, between our study groups. In the studies by Martínez-Llordella et al., 2007 and Martínez-Llordella et al., 2008 in which peripheral blood gene expression was studied, as well as in another study (29), the circulating Vδ1/Vδ2 γδT-cell ratio was higher in tolerant LTx recipients compared to control or non-tolerant LTx recipients, and was suggested to be a marker for tolerance. However, we (21) and others (17, 20) have shown that a higher Vδ1/Vδ2 γδT-cell ratio is associated with CMV latency in LTx recipients. Recently, we also demonstrated that the Vδ1/Vδ2 γδT-cell ratio in peripheral blood does not differ between TOL and CTRL LTx recipients matched for CMV serostatus (30). Therefore it is likely that in these previous studies the tolerant group of LTx recipients harbored more CMV seropositive LTx recipients than the control or non-tolerant groups of LTx recipients. Hence, the different gene expression of SLAMF7 and NKG7 found by Martínez-Llordella et al., 2008 (10) could be rather suitable for identification of CMV positive LTx recipients than tolerant LTx recipients.

In our study relative gene expression of Treg related markers TET1, TET2, NRP1 and HELIOS did not differ in TOL LTx recipients compared to all other study groups, whereas FOXP3 expression was significantly influenced by the use of IS. Although previously higher expression of FOXP3 in blood of tolerant LTx recipients without IS compared to non-tolerant LTx recipients after reintroduction of IS regimen was reported, this difference was not observed when FOXP3 was measured before IS weaning (12, 13), supporting our observation that FOXP3 expression is suppressed by IS. In these previous studies, peripheral blood expression of TET2 and NRP1 were similar in tolerant and non-tolerant LTx recipients, comparable to our own data. HELIOS expression was reported to be enhanced in tolerant LTx recipients, but this difference was not observed before IS weaning (12, 13). Together with the other gene expression data this shows that the assessed markers related to Tregs, NK cells or γδT-cells are rather influenced by prior CMV infection and/or use of IS, than indicative of operational tolerance.

The strength of our study is that we carefully matched TOL, CTRL, MIN and HC study groups for important clinical parameters known to influence circulating immune cells and their gene expression, and thereby eliminated potential confounders. Furthermore, the selected forward and reverse primer pairs were thoroughly designed and tested for their optimal annealing temperature and amplification efficiency and specificity. This resulted in exclusion of ERBB2 and UBD genes. Another strength is that we used three references genes to study the relative gene expression of selected genes, in contrast to other studies that only used one reference gene (10–12), making our data more robust. Moreover, in our study we clearly state which splice variants of selected genes were measured for full disclosure and maximum transparency. Lastly, in contrast to other studies in this field, we used appropriate statistical analyses with correction for multiple testing to analyze our data. Our study also has some limitations. A weaker part of our study is that we included CTRL LTx recipients with regular IS regimen with unknown tolerance status to compare to tolerant LTx recipients without any IS regimen. However, it is expected that the majority of CTRL LTx recipients are non-tolerant towards their graft. Unfortunately, we did not have access to samples of TOL LTx recipients before IS weaning, which would have facilitated a better comparison of gene expression profiles between the different groups. To account for the difference in IS use, we included a minimal IS regimen group and a healthy control group. The small study group sizes in this study are another weakness, although this is not unusual when studying operational tolerance in LTx recipients. As in other recently published studies on operational tolerance (6, 7), liver function tests were used as an indicator of tolerance instead of protocol liver biopsies due to possible complications that could arise. The lack of protocol biopsies in our study may result in an absence of diagnoses of subclinical rejection. Subclinical rejection may also impact peripheral blood gene expression profiles. However, the clinical implications of subclinical rejection and a possible relation to graft damage are still unclear (31–33).

Here we studied peripheral blood expression of Treg, NK cell and γδT-cells related genes described in previous studies that identified operational tolerance amongst LTx recipients. Unfortunately, we could not confirm their capacity to discriminate tolerant LTx recipients from control LTx recipients. Instead, we found a confounding effect of IS usage and prior CMV infection, on expression of many selected genes. In the future whole genome RNA sequencing should be performed on PBMCs of carefully matched tolerant and non-tolerant LTx recipients before IS weaning to identify a tolerance predicting gene expression profile suitable for selecting recipients eligible for prospective IS weaning. This gene expression profile should be also be validated at the level of protein to confirm its importance.

## Data Availability Statement

The data discussed in this publication have been deposited in NCBI's Gene Expression Omnibus and are accessible through GEO Series accession number GSE192989 (https://www.ncbi.nlm.nih.gov/geo/query/acc.cgi?acc=GSE192989).

## Ethics Statement

The studies involving human participants were reviewed and approved by Medical Ethics committee Erasmus MC. The patients/participants provided their written informed consent to participate in this study.

## Author Contributions

AD participated in research design and data extraction, performance of the research and prepared the first and subsequent drafts of the manuscript for submission. MG participated in data acquisition and extraction. PB and LN participated in research design and data extraction. RK provided critical input for writing of the manuscript. MP provided critical insight of the research. MB provided critical input for writing of the manuscript and critical insight of the research. NL and JK obtained funding, provided critical input for writing of the manuscript and critical insight of the research. All authors approved the final version of the manuscript.

## Funding

This work was supported by an Erasmus MC PhD-grant (MRACE) 2015.

## Conflict of Interest

The authors declare that the research was conducted in the absence of any commercial or financial relationships that could be construed as a potential conflict of interest.

## Publisher’s Note

All claims expressed in this article are solely those of the authors and do not necessarily represent those of their affiliated organizations, or those of the publisher, the editors and the reviewers. Any product that may be evaluated in this article, or claim that may be made by its manufacturer, is not guaranteed or endorsed by the publisher.

## Abbreviations

Aza, azathioprine; CMV, cytomegalovirus; CsA, cyclosporine A; CTRL, control LTx recipients; HC, healthy controls; IS, immunosuppressive drugs; LTx, liver transplantation; MIN, minimal IS regimen LTx recipients; MMF, mycophenolate mofetil; NK cells, natural killer cells; PBMCs, peripheral blood mononuclear cells; PCR, polymerase chain reaction; Pred, prednisolone; Tac, tacrolimus; TOL, tolerant LTx recipients; Tregs, regulatory T-cells.
