# Supplementary material for: Current Tolerance-Associated Peripheral Blood Gene Expression Profiles After Liver Transplantation Are Influenced by Immunosuppressive Drugs and Prior Cytomegalovirus Infection
Source: Front Immunol. 2022 Jan 11;12:738837. doi: 10.3389/fimmu.2021.738837 (PMC8787265; doi:10.3389/fimmu.2021.738837)
Supplement: Supplementary file 1 [file DataSheet_1.docx]

## Supplementary material

**Supplementary Table 1 Analyzed genes with their transcript variants and forward and reverse primer sequences.**

| Gene | Other nomenclature | Number of splice variants | Name in graph | Forward primer | Reverse primer | Accession number | Splice variant |
| --- | --- | --- | --- | --- | --- | --- | --- |
| *Housekeeping genes* | | | | | | | |
| GADPH | G3PD; GAPD; HEL-S-162eP | 8 | NA | AAGGTCGGAGTCAACGGATTT | ACCAGAGTTAAAAGCAGCCCTG | NM_002046.7 | Transcript variant 1 |
|  |  |  |  |  |  | NM_001289745.3 | Transcript variant 3 |
|  |  |  |  |  |  | NM_001289746.2 | Transcript variant 4 |
|  |  |  |  |  |  | NM_001357943.2 | Transcript variant 7 |
| GUSB | BG; MPS7 | 4 | NA | CAGGTGATGGAAGAAGTGG | GTTGCTCACAAAGGTCACAG | NM_000181.4 | Transcript variant 1 |
|  |  |  |  |  |  | NM_001284290.2 | Transcript variant 2 |
|  |  |  |  |  |  | NM_001293104.2 | Transcript variant 3 |
|  |  |  |  |  |  | NM_001293105.2 | Transcript variant 4 |
| HPRT1 | HPRT; HGPRT | 1 | NA | GCTATAAATTCTTTGCTGACCTGCTG | AATTACTTTTATGTCCCCTGTTGACTGG | NM_000194.3 | NA |
| *NK-cell related function* | | | | |  |  |  |
| KLRB1 | NKR; CD161; CLEC5B; NKR-P1; NKRP1A; NKR-P1A; hNKR-P1A | 1 | KLRB1 | CTGGGTTGAGTGTTTCAGTGAC | TTTCTCTCGGAGTTGCTGCC | NM_002258.2 | NA |
| CD160 | NK1; BY55; NK28 | 1 | CD160 | AAGGTCTGGGTAATGCTGGTC | AGACTCATCTTGCTGTAGCTGT | NM_007053.3 | NA |
| KLRC4 | NKG2F; NKG2-F | 1 | KLRC4 | CACTGCAAAGGTTTACTGCCAC | GTTCTGCTCCAGTACTCCAATACA | NM_013431.2 | NA |
| KLRF1 | NKp80; CLEC5C | 4 | KLRF1 | GGAGTTCTGCCCAAACATCTC | TGGCATAGTACCAACAGGATCAA | NM_001291822.1 | Transcript variant 1-s |
| NKG7 | GIG1; GMP-17; p15-TIA-1 | 2 | NKG7-1 | TTCTGGTTTGAGGCTGTGGG | GTCACGTGGATGTAGCCTGATA | NM_005601.4 | Transcript variant 1 |
|  |  |  | NKG7-2 | GGACATCATATCAGGCCACG | CTGGGGACAAGGACAAGAGAG | NM_001363693.1 | Transcript variant 2 |
| *Treg-related function* | | | | | | | |
| TET1 | LCX; CXXC6; bA119F7.1 | 1 | TET1 | ACTGCCAACCTTAGGGAGTAAC | ATGCCTCTTTCACTGGGTGAG | NM_030625.3 | NA |
| TET2 | MDS; IMD75; KIAA1546 | 2 | TET2 | TTACAACGCTTGGAAGCAGG | CTAGTTGAATTCAGCAGCTCAG | NM_001127208.2 | Transcript variant 1 |
|  |  |  |  |  |  | NM_017628.4 | Transcript variant 2 |
| NRP1 | NP1; NRP; BDCA4; CD304; VEGF165R | 6 | NRP1 | AGCACCGAGAGAACAAGGTG | CCGCAGCTCAGGTGTATCAT | NM_003873.6 | Transcript variant 1 |
|  |  |  |  |  |  | NM_001024628.2 | Transcript variant 2 |
|  |  |  |  |  |  | NM_001024629.2 | Transcript variant 3 |
|  |  |  |  |  |  | NM_001244972.1 | Transcript variant 4 |
|  |  |  |  |  |  | NM_001244973.1 | Transcript variant 5 |
|  |  |  |  |  |  | NM_001330068.1 | Transcript variant 7 |
| FOXP3 | JM2; AIID; IPEX; PIDX; XPID; DIETER | 2 | FOXP3 | CAAGTTCCACAACATGCGACC | GAAGGCAAACATGCGTGTGAA | NM_014009.3 | Transcript variant 1 |
|  |  |  |  |  |  | NM_001114377.2 | Transcript variant 2 |
| IL2RB | CD122; IMD63; IL15RB; P70-75 | 1 | IL2RB | TGGAGAGATGGCCACGGT | TTACATCCACAGGGTGGAGC | NM_000878.5 | NA |
| HELIOS | ANF1A2; IKZF2; ZNF1A2; ZNFN1A2 | 7 | HELIOS | GGAAACAGAGGCTATTGATGGCT | TGTCCATTGGGTGTGCTTGA | NM_016260.3 | Transcript variant 1 |
|  |  |  |  |  |  | NM_001079526.2 | Transcript variant 2 |
|  |  |  |  |  |  | NM_001371274.1 | Transcript variant 3 |
|  |  |  |  |  |  | NM_001371275.1 | Transcript variant 4 |
|  |  |  |  |  |  | NM_001371276.1 | Transcript variant 5 |
|  |  |  |  |  |  | NM_001371277.1 | Transcript variant 6 |
|  |  |  |  |  |  | NM_001387220.1 | Transcript variant 7 |
| SMAD2 | JV18; MADH2; MADR2; JV18-1; hMAD-2; hSMAD2 | 3 | SMAD2 | CCGACACACCGAGATCCTAAC | GAGGTGGCGTTTCTGGAATATAA | NM_005901.6 | Transcript variant 1 |
|  |  |  |  |  |  | NM_001003652.4 | Transcript variant 2 |
|  |  |  |  |  |  | NM_001135937.2 | Transcript variant 3 |
| SMAD3 | LDS3; LDS1C; MADH3; JV15-2; HSPC193; HsT17436 | 4 | SMAD3 | TGGACGCAGGTTCTCCAAAC | CCGGCTCGCAGTAGGTAAC | NM_005902.4 | Transcript variant 1 |
|  |  |  |  |  |  | NM_001145102.1 | Transcript variant 2 |
|  |  |  |  |  |  | NM_001145103.1 | Transcript variant 3 |
|  |  |  |  |  |  | NM_001145104.1 | Transcript variant 4 |
| *Other* | | | | | | | |
| IRF5 | SLEB10 | 7 | IRF5 | AGGGCTTCAATGGGTCAACG | ACGCCTTCGGTGTATTTCCC | NM_032643.4 | Transcript variant 2 |
|  |  |  |  |  |  | NM_001098627.3 | Transcript variant 3 |
|  |  |  |  |  |  | NM_001098629.3 | Transcript variant 5 |
|  |  |  |  |  |  | NM_001098630.3 | Transcript variant 6 |
|  |  |  |  |  |  | NM_001242452.3 | Transcript variant 8 |
|  |  |  |  |  |  | NM_001347928.2 | Transcript variant 9 |
|  |  |  |  |  |  | NM_001364314.2 | Transcript variant 10 |
| EGR2 | CHN1; AT591; CMT1D; CMT4E; KROX20 | 5 | EGR2 | CCATCTTTCCCAATGCCGAAC | GGGAGATCCAACGACCTCTTC | NM_000399.5 | Transcript variant 1 |
|  |  |  |  |  |  | NM_001136177.3 | Transcript variant 2 |
|  |  |  |  |  |  | NM_001136178.1 | Transcript variant 3 |
|  |  |  |  |  |  | NM_001136179.3 | Transcript variant 4 |
|  |  |  |  |  |  | NM_001321037.2 | Transcript variant 5 |
| CXCL8 | IL8; NAF; GCP1; LECT; LUCT; NAP1; GCP-1; LYNAP; MDNCF; MONAP; NAP-1; SCYB8 | 2 | CXCL8 | GAAACCACCGGAAGGAACCAT | AAACTGCACCTTCACACAGAGC | NM_000584.4 | Transcript variant 1 |
|  |  |  |  |  |  | NM_001354840.2 | Transcript variant 2 |
| ZBTB21 | ZNF295 | 5 | ZBTB21-1 | TGAAGACCGATAAACTCAAGCCA | CTGTCCTTTGAGACGCTCCT | NM_020727.5 | Transcript variant 2 |
|  |  |  |  |  |  | NM_001320729.2 | Transcript variant 4 |
|  |  |  | ZBTB21-2 | TCGAGACTGAAGACCGAAGAC | CACTTTGATCCTCGCACACA | NM_001098402.2 | Transcript variant 1 |
|  |  |  |  |  |  | NM_001098403.2 | Transcript variant 3 |
|  |  |  |  |  |  | NM_001320731.2 | Transcript variant 5 |
| CX3CR1 | V28; CCRL1; GPR13; CMKDR1; GPRV28; CMKBRL1 | 4 | CX3CR1-1 | TCTTCCACCATGAGCAGGC | GTGAAGGCCTCTAGTCGCTG | NM_001171171.1 | Transcript variant 2 |
|  |  |  | CX3CR1-2 | CAGAGGTTCCCTTGGCAGTC | TCCCAAAGACCACGATGTCC | NM_001337.3 | Transcript variant 4 |
| OSBPL5 | ORP5; OBPH1 | 3 | OSBPL5 | ATCACCCTCATCGCTCTGTG | GGAAGACCCGTTCAGTGGG | NM_020896.4 | Transcript variant 1 |
|  |  |  |  |  |  | NM_145638.3 | Transcript variant 2 |
|  |  |  |  |  |  | NM_001144063.2 | Transcript variant 3 |
| SLAMF7 | 19A; CS1; CD319; CRACC | 10 | SLAMF7-1 | ACAGAGTACGACACAATCCCTC | TTGGTGTGTCTGGCATCGTG | NM_021181.5 | Transcript variant 1 |
|  |  |  |  |  |  | NM_001282589.1 | Transcript variant 3 |
|  |  |  |  |  |  | NM_001282590.2 | Transcript variant 4 |
|  |  |  |  |  |  | NM_001282591.1 | Transcript variant 5 |
|  |  |  |  |  |  | NM_001282594.1 | Transcript variant 8 |
|  |  |  |  |  |  | NM_001282595.1 | Transcript variant 9 |
|  |  |  | SLAMF7-2 | CAGTGGCTGACTTCCAGAGAG | GACCAGCTCTTTCACGGGTC | NM_021181.5 | Transcript variant 1 |
|  |  |  |  |  |  | NM_001282588.1 | Transcript variant 2 |
|  |  |  |  |  |  | NM_001282589.1 | Transcript variant 3 |
|  |  |  |  |  |  | NM_001282592.2 | Transcript variant 6 |
|  |  |  | SLAMF7-3 | GTGGCTTCATTTCAGTGGCTG | CTTTGACAGGTGCTCTGTGAG | NM_001282590.2 | Transcript variant 4 |
|  |  |  |  |  |  | NM_001282591.1 | Transcript variant 5 |
|  |  |  |  |  |  | NM_001282593.1 | Transcript variant 7 |
|  |  |  |  |  |  | NM_001282594.1 | Transcript variant 8 |
|  |  |  |  |  |  | NM_001282596.2 | Transcript variant 10 |


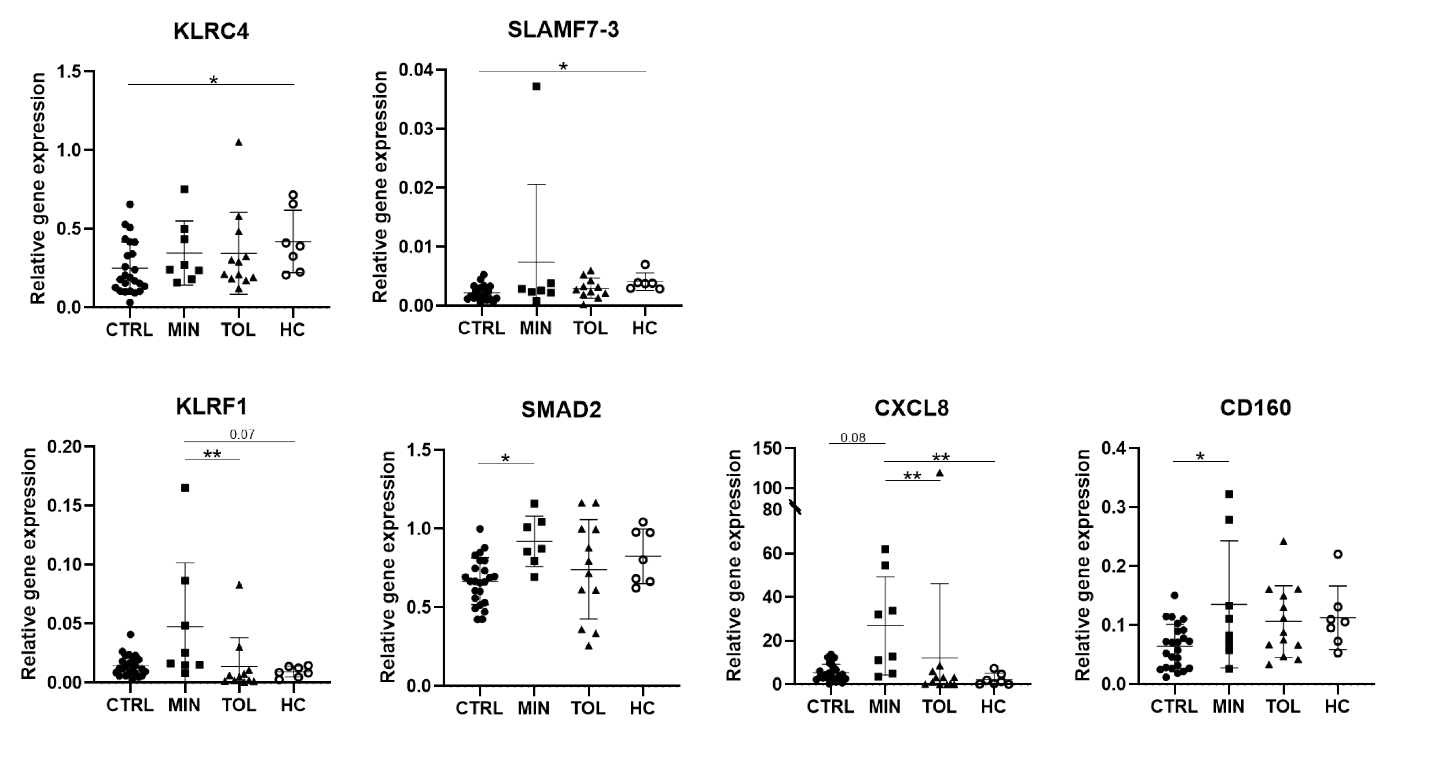


**Supplementary Figure 1 Relative expression of genes differentially expressed between several study groups.** Differentially expressed KLRC4, SLAMF7-3, KLRF1, SMAD2, CXCL8 and CD160 in CTRL, MIN, TOL and HC groups are depicted. * P<0.05, ** P<0.01 CTRL, control LTx recipients; HC, healthy control; LTx, liver transplantation; MIN, minimal IS regimen LTx recipients; TOL, tolerant LTx-recipients.
